# CD24 induces changes to the surface receptors of B cell microvesicles with variable effects on their RNA and protein cargo

**DOI:** 10.1038/s41598-017-08094-8

**Published:** 2017-08-17

**Authors:** D. Craig Ayre, Ian C. Chute, Andrew P. Joy, David A. Barnett, Andrew M. Hogan, Marc P. Grüll, Lourdes Peña-Castillo, Andrew S. Lang, Stephen M. Lewis, Sherri L. Christian

**Affiliations:** 1 Department of Biochemistry, Memorial University of Newfoundland, St. John’s, Newfoundland and Labrador Canada; 2 Atlantic Cancer Research Institute, Moncton, New Brunswick Canada; 3 Departments of Biology, Memorial University of Newfoundland, St. John’s, Newfoundland and Labrador Canada; 4 Department of Computer Science, Memorial University of Newfoundland, St. John’s, Newfoundland and Labrador Canada; 5 Department of Microbiology & Immunology, Dalhousie University, Halifax, Nova Scotia Canada; 6 Department of Biology, University of New Brunswick, Saint John, New Brunswick Canada; 7 Department of Chemistry & Biochemistry, Université de Moncton, Moncton, New Brunswick Canada

## Abstract

The CD24 cell surface receptor promotes apoptosis in developing B cells, and we recently found that it induces B cells to release plasma membrane-derived, CD24-bearing microvesicles (MVs). Here we have performed a systematic characterization of B cell MVs released from WEHI-231 B lymphoma cells in response to CD24 stimulation. We found that B cells constitutively release MVs of approximately 120 nm, and that CD24 induces an increase in phosphatidylserine-positive MV release. RNA cargo is predominantly comprised of 5S rRNA, regardless of stimulation; however, CD24 causes a decrease in the incorporation of protein coding transcripts. The MV proteome is enriched with mitochondrial and metabolism-related proteins after CD24 stimulation; however, these changes were variable and could not be fully validated by Western blotting. CD24-bearing MVs carry Siglec-2, CD63, IgM, and, unexpectedly, Ter119, but not Siglec-G or MHC-II despite their presence on the cell surface. CD24 stimulation also induces changes in CD63 and IgM expression on MVs that is not mirrored by the changes in cell surface expression. Overall, the composition of these MVs suggests that they may be involved in releasing mitochondrial components in response to pro-apoptotic stress with changes to the surface receptors potentially altering the cell type(s) that interact with the MVs.

## Introduction

Extracellular vesicles (EVs) are a collection of membrane-enclosed structures released from cells, broadly defined into three major sub-types: exosomes, microvesicles (MVs), and apoptotic bodies^1^. Exosomes are 50 to 100 nm-sized vesicles that are released from multi-vesicular bodies within the cytosol^2^. MVs (also termed ectosomes, shedding vesicles or microparticles) are 100 to 1000 nm-sized vesicles that bud directly from the plasma membrane^2^. Lastly, apoptotic bodies are larger vesicles (1–5 μm) that result from membrane blebbing in the final stages of apoptosis^3^. The production of EVs is ubiquitous, having been identified from many cell types, and isolated from virtually all body fluids^1^. Thus, EV production likely represents an innate, basal cellular process to serve as a cell - cell communication vehicle to influence local, or potentially even distal, recipients.

EVs can influence recipient cells through a variety of means. One important mediator is through the delivery of mRNA and miRNA from donor to recipient cells. For example, adipocyte EVs are capable of upregulating lipogenesis in recipient cells via the transfer of RNA^4^. *In vitro* assays have also demonstrated the ability of EVs to transfer bio-active miRNA (such as miR-335) to silence specific target mRNAs in recipient cells^5^, a property that has recently been exploited to deliver mutant KRAS-silencing siRNA and shRNAs^6^. EV transfer of the GPI-anchored proteins CD55 and CD59 to erythrocytes can correct paroxysmal nocturnal hemoglobinuria by inhibiting complement-mediated red blood cell lysis^7, 8^. During immune responses, EVs are known to participate in the transfer of antigens to professional antigen-presenting cells, or to carry specific immuno-modulatory cytokines^9^. EVs can also affect the growth and development of cancers. Mouse fibroblasts expressing the oncogenic diffuse B cell lymphoma gene promote the growth and survival of untransformed cells via the EV-mediated transfer of focal adhesion kinase (FAK) protein^10^. It is therefore clear that EV cargoes, including mRNA, miRNA, luminal, and surface proteins, allow EVs to alter the biology of recipient cells.

CD24, also called Heat Stable Antigen (HSA), is a glycophosphatidylinositol (GPI)-linked protein expressed on the surface of numerous cell types that is post-translationally modified with a dense and variable network of N- and O-linked glycosylations^11^. One of the most well-described effects of CD24-mediated signalling is its promotion of apoptosis in immature and developing B cells^12–15^. Recently, we have shown that in addition to promoting apoptosis, stimulation of CD24 via antibody (Ab)-mediated crosslinking to mimic ligand binding is associated with the release of plasma membrane-derived MVs from *ex vivo* bone marrow-derived B cells and the mouse WEHI-231 B cell lymphoma cell line^15^. While CD24 has been shown to be present on EVs derived from amniotic fluid and urine^16^, this was the first report of CD24 stimulation directly promoting EV production. Further analysis revealed that CD24 itself was enriched in EVs released from WEHI-231 cells following CD24 stimulation; however, no analysis of EV cargo has been performed following CD24 stimulation in any model system, nor has the contribution of CD24 stimulation to EV generation been described.

Therefore, the objective of this study was to isolate and characterize the EVs released in response to CD24 stimulation. We have used the WEHI-231 B cell line for this first characterization because this is the only model system where CD24-mediated MV release has been extensively validated^15^. Using a combination of morphology, RNA-Seq, proteomics, and flow cytometry we have firmly established that CD24 stimulation promotes MV and not exosome release. We also found that the RNA cargo and the MV proteome are relatively stable in response to stimulation, but that surface receptor composition is regulated so that the MV receptor composition is distinct from the cell surface. Overall, our data show that B cells constitutively release MVs, but that CD24 signalling affects the surface composition in a manner that does not reflect their cellular origin, suggesting a regulated system for vesicle packaging.

## Results

### EV released from isotype and anti-CD24 stimulated WEHI-231 cells are morphologically similar

**Figure 1:**
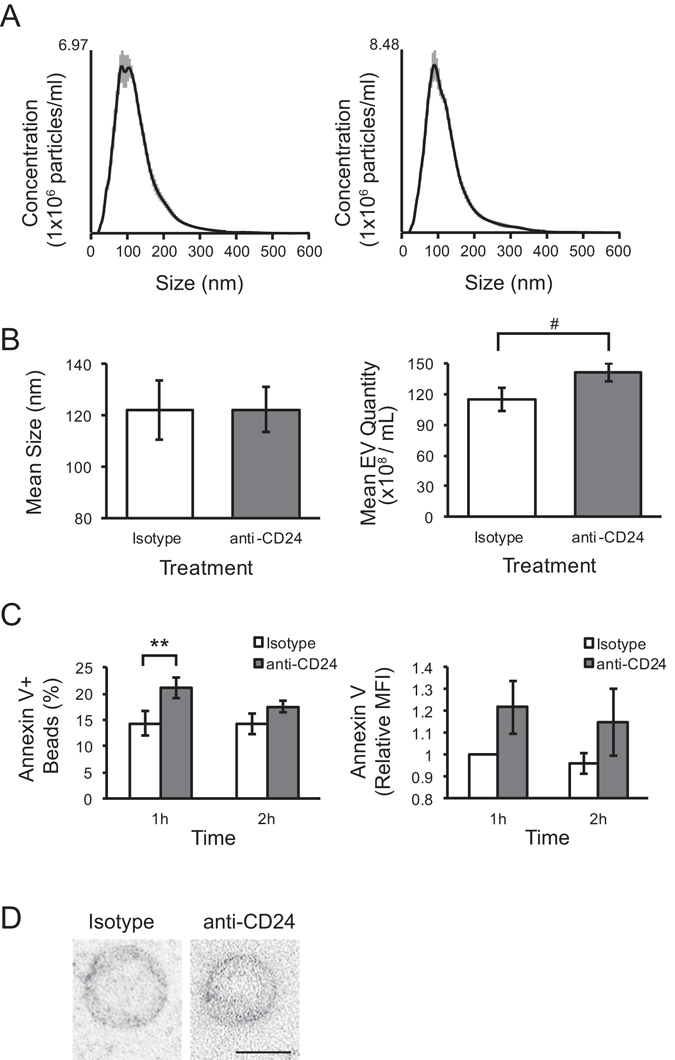
The quantification and morphology of MVs released from B cells with and without CD24 stimulation. (**A**) Representative nanoparticle tracking (NTA) plots of particle sizes and concentrations in supernatants from cells stimulated with either isotype (right) or anti-CD24 (left) Ab after 1 h. Grey lines show ± 1 standard deviation of the mean in measurements. n = 3 biological replicates with 5 technical replicates each. (**B**) The mean size (left) and concentration (right) ± SEM of particles from supernatants analyzed by NTA, n = 3. Statistical significance was assessed using Students’ paired t-test, ^#^p = 0.08. (**C**) Mean % positive (left) and relative mean fluorescent intensity (MFI; right) of Annexin V-FITC beads used to capture CD24-bearing MVs released from isotype or anti-CD24 stimulated cells for the indicated times, n = 3. Statistics assessed by a two-tailed Student’s paired t-test. **p < 0.01. (**D**) Representative transmission electron microscopy (TEM) images of Vn96-isolated EVs from cells stimulated with either isotype or anti-CD24 for 1 h. Scale bar = 100 nm.

Previously, we found that Ab-mediated stimulation of CD24 induced the formation of CD24-bearing EVs from B cells, which we concluded were plasma-membrane derived MVs^15^. Here we further characterized the size, shape and quantity of the EVs released by WEHI-231 cells after anti-CD24 stimulation compared to isotype control treatment (Fig. 1).

Particles of MV size were detected using nanoparticle tracking analysis (NTA) following 1 h stimulation in both conditions (Fig. 1A). The mean size from isotype-treated samples was 122 +/− 54 nm, and from anti-CD24 treated samples was 122 +/− 56 nm (Fig. 1B left), consistent with the upper range of exosomes and the lower range of MVs^2^. Isotype-treated cell supernatant contained an average of 114.7 × 10^8^ particles/mL, whereas anti-CD24 stimulated cell supernatant contained an average of 141.3 × 10^8^ particles (Fig. 1B, right). While not reaching statistical significance, each supernatant from CD24 treated cells contained more particles than the matching isotype supernatant, and there was an overall trend towards increased particle numbers following CD24 stimulation (p = 0.07).

We next quantified the population of CD24-bearing EVs that express phosphatidylserine by flow cytometric-detection of Annexin V on CD24+ EVs captured on streptavidin-coated magnetic beads. While individual EVs cannot be analyzed using this approach, we found a statistically significant increase in the number of Annexin V+ beads in response to 1 h anti-CD24 stimulation (Fig. 1C left) with 14.3 ± 2.3% of beads from isotype-treated samples, and 21.2 ± 2.0% of beads from anti-CD24 treated samples being Annexin V+ (p = 0.009). After 2 h of anti-CD24 stimulation, the number of Annexin V+, bead-captured EV was comparable to that of isotype treatment indicating that the promotion of Annexin V+ EV formation by CD24 was transitory.

We isolated EVs from both isotype and anti-CD24 stimulated cells for transmission electron microscopy (TEM) analysis using Vn96 peptide-based capture, as previously validated^17^. We isolated round structures consistent with both the size estimates from the NTA and our previous results using FACS-based bead sizing that showed that CD24 was associated with EVs smaller than 200 nm in diameter (Fig. 1D)^15^. There was no difference in morphology between EVs isolated from isotype or anti-CD24 stimulated cells.

Together these results indicate that B cells constitutively release EVs that are approximately 120 nm in diameter and these vesicles do not vary in their size or morphology following CD24 stimulation; however, there is a statistically significant increase in the amount of phosphatidylserine-positive EVs, indicative of MVs, following 1 h of anti-CD24 stimulation that does not persist over time.

### Individual transcripts are not preferentially packaged but overall protein coding transcripts are reduced in EVs in response to CD24 stimulation

**Figure 2:**
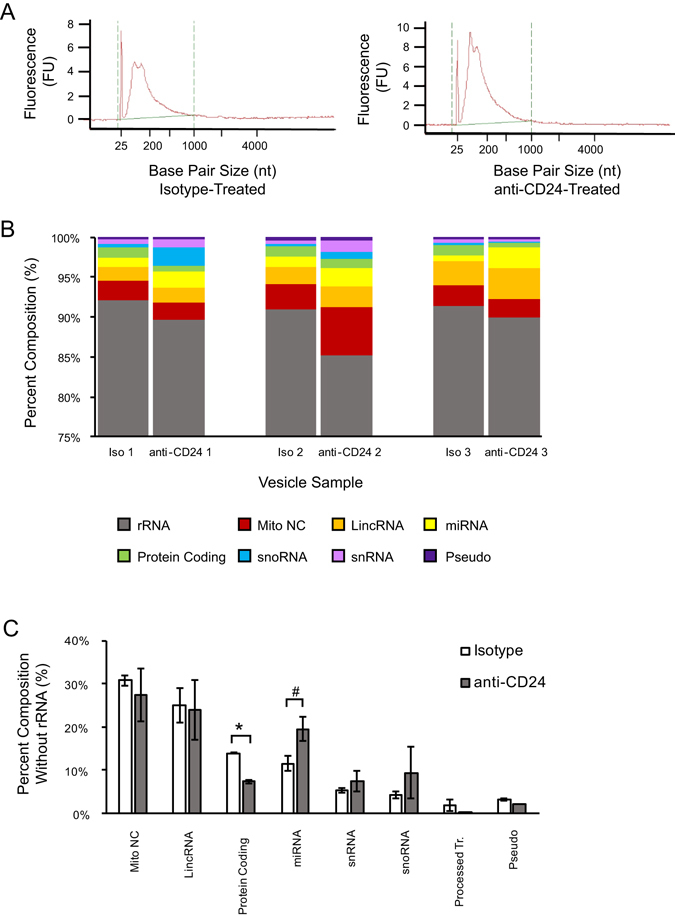
CD24 stimulation alters the abundance of protein coding transcripts loaded into B cell MVs. (**A**) Representative RNA size distributions of MVs collected from cells stimulated with either isotype (right) or anti-CD24 (left) Ab after 1 h. (**B**) The RNA incorporated into MVs from cells after 1 h of either isotype or anti-CD24 Ab stimulations categorized into one of 8 sub-categories containing >0.2% of the total RNA, n = 3 biological replicates. (**C**) The mean ± SEM percentage of the 8 major non-rRNA transcripts with greater than 2% of RNA abundance from MVs isolated from either isotype (white bars) or anti-CD24 (grey bars) stimulated cells. n = 3, statistics were assessed using a two-tailed Student’s t-test, ^#^p = 0.07; *p < 0.05.

We next used RNA-seq to characterize the RNA carried by EVs released by WEHI-231 cells. We found that both isotype and anti-CD24 stimulated cells produce EVs that carry RNA of approximately 200 nucleotides, consistent with other reports of RNA isolated from MVs (Fig. 2A)^18^. As there was no evidence of 18S or 28S RNA by Bioanalyzer analysis of the EV population, we sequenced all EV RNA without rRNA depletion.

We did not identify any individual transcripts that were differentially incorporated between EVs from isotype or anti-CD24 stimulated cells. Surprisingly, we found that 89.5% of the transcripts in all 6 EV samples were annotated as either rRNA or ribozyme by BioMART^19^. The majority (95.2%) of these transcripts were annotated as 5.8S (72.7%) and 5S (22.5%) rRNA, (Fig. 2B). The RNA carried by EVs is strongly influenced by their sub-type and cell of origin; however, the lack of 18S and 28S, but presence of 5/5.8S rRNA, is consistent with other reports on MV RNA^18, 20^. Of the remaining transcripts, approximately 29.1 ± 2.9% (3.1% of the total RNA) were mitochondrial non-coding mt-rRNA and mt-tRNA (Fig. 2C).

Interestingly, we found a statistically significant decrease in the total number of protein-coding transcripts in EVs from CD24-stimulated cells (p = 0.002). Of the top 50 protein coding transcripts, 14 were mitochondrial genes. There was also a trend towards increased abundance of miRNA transcripts in the EVs (p = 0.08) (Fig. 2C). Even though our analysis did not identify any individual statistically significant differentially expressed transcripts, these data show there are changes in the overall distribution of RNA incorporated into these EVs.

We next performed gene ontology (GO) enrichment analysis on the 50 most abundant protein coding transcripts using ToppFun (Supplemental File 1). These were enriched for 16 Biological Process (BP) terms, 13 Molecular Function (MF) terms and 9 Cellular Component (CC) terms, at a Bonferroni-corrected P-value (q-value) of q = 0.05 (Supplemental File 2). Using Revigo, we visualized the associations between GO terms based on their associations to one another (Supplemental Fig. 1). The BP terms belonged to one of two major groups: Electron transport chain and the Generation of precursor metabolites and energy. Similarly, four of the five MF groups belonged to mitochondrial-associated functions: NADH dehydrogenase activity, Hydrogen ion transmembrane transporter activity, Oxioreductase activity, and Electron carrier activity. Finally, the CC terms were primarily associated as Mitochondrial inner membrane, and Respiratory chain. Overall, these data demonstrate that protein-coding transcripts in EVs released by these B cells are associated with mitochondrial functions, regardless of CD24 stimulation.

Finally, across the six samples, we found there were on average 22 distinct miRNA transcripts, of which 12 were common to all EVs. Only two, miR-6236 and miR-5099, were annotated and there was a trend towards increased incorporation in these transcripts in EVs from anti-CD24 stimulated cells (p = 0.09 for both). As the remainder of the common transcripts were unannotated or predicted, no functional enrichment analysis could be performed.

### CD24 stimulation may enrich specific proteins in the EV cargo of WEHI-231 cells

We next used mass spectrometry (MS; specifically, nanoLC-MS/MS) to analyze the protein cargo in Vn96-isolated EVs from both isotype and anti-CD24 stimulated cells. We found that the EVs carried a total of 460 unique proteins, detected at the 2-peptide cut-off level per protein (Supplemental File 1). We found 58 proteins, which mapped to 41 annotated genes, that were common to EVs from both conditions (Supplemental File 1). There was considerable heterogeneity among the EV samples, with no peptides that uniquely distinguished EVs released by isotype-treated cells from anti-CD24 treated cells. However, 79 proteins (mapping to 77 annotated genes) were found in two or more of the anti-CD24 stimulated EVs, but present in only a single EV sample from isotype-treated cells (Supplemental File 1), suggesting these proteins may be preferentially enriched into EVs by CD24. We did not observe the reciprocal effect of peptides preferentially enriched into EVs from isotype-treated cells. Finally, 153 peptides were detected in only one of the 6 replicates, suggesting they are incorporated into B cell EVs at low abundance, or randomly.

**Figure 3:**
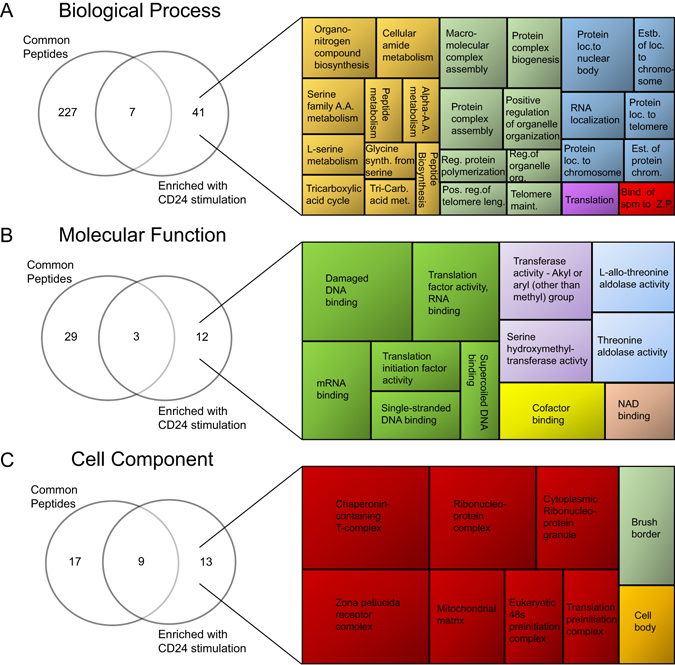
Proteomics analysis suggests that CD24 stimulation causes enrichment of proteins from specific functional categories into MVs. Gene Ontology (GO) enrichment analysis was performed on proteins common to MVs from all 6 isotype and anti-CD24 samples in comparison to the proteins enriched in MVs after 1 h anti-CD24 stimulation (Supplemental File 1). Venn diagrams (left panels) show GO terms associated with all MVs or those associated with the proteins enriched in MVs after anti-CD24 stimulation. The numbers indicate the number of unique GO terms associated with the respective protein lists. Enriched GO terms were visualized by Revigo^70^ (right panels). (**A**) CD24-enriched biological process (BP): Cellular amide metabolism (yellow), macromolecular complex assembly (green) and protein localization to nuclear body (blue). (**B**) CD24-enriched molecular functions (MF): Damaged DNA binding (green), transferase activity (purple) and threonine aldolase activity (blue). (**C**) CD24-enriched cellular component annotations were primarily grouped as chaperonin-containing T-complex (red). A.A. = amino acid, Synth = synthesis, Carb = carboxylic, Reg = regulation, Pos. = positive, Leng. = length, org = organization, Maint = maintenance, Loc = localization, Est = Establishment, Spm = sperm, Z.P = zona pellucida.

We used Toppfun to identify the biological functions of proteins enriched in EVs from CD24-stimulated cells, by identifying their unique GO terms for the 41 common EV proteins. We found that proteins enriched in EVs from CD24-stimulated cells were associated with 41 BP (Fig. 3A), 12 MF (Fig. 3B) and 13 CC terms (Fig. 3C). We identified five major GO associations within the 41 BP, with most terms categorized as Cellular amide metabolism, Macromolecular complex assembly, or Protein localization to nuclear body (Fig. 3A). Similarly, we found three major associations in MF ontologies, with the largest two being Damaged DNA binding and Transferase activity related to one carbon metabolism (Fig. 3B). Finally, the CC terms were primarily categorized as Chaperonin-containing T complex, or other protein-binding complexes (Fig. 3C), which function to regulate protein-protein interactions^21^ and protein folding. Overall, as with the transcript analysis, there was a strong association between EV protein cargo, and mitochondrial or metabolic functions; however, we do find other associations that suggest the regulation of protein localization or protein complexes.

We also identified associations with CD24-EV enriched proteins to published proteomics and pathway data (Supplemental File 2). There were significant correlations with four different publications that examine exosome-associated protein cargo from B cells, podocytes, prostrate secretions and urine^22–25^. The most significant pathway associations involved metabolism-related functions, such as carbon metabolism (q = 4.95 × 10^–6^), serine/glycine biosynthesis (q = 3.9 × 10^−4^), TCA cycle (q = 3.9 × 10^−4^) and conversion of glucose to acetyl CoA (q = 3.9 × 10^−4^), supporting the identified associations with mitochondrial-related functions.

To validate the proteomics data, we selected five proteins for Western blot analysis. Owing to the heterogeneity in the MS data, we again selected proteins found in two or more anti-CD24 stimulated EV samples, but present in no more than one sample from isotype-treated cells, with each protein having different biological associations (Table 1). We examined the expression of these proteins in both isotype or anti-CD24 stimulated cell lysates, and their respective Vn96-isolated EVs. Heat Shock Protein (HSP) 90 was used as a loading control, as it is ubiquitously expressed in cells and was present in all six EV samples we analysed by MS.

**Table 1:** Selected proteins from mass spectrometry data for validation by Western blot.

Protein Name	Symbol	Rationale
Heat shock protein 90	HSP90	Ubiquitously expressed in cells, and present in all EVs. Used as Western blot loading control^17^.
Serine hydroxymethyltransferase 2 (mitochondrial)	SHMT2	Enrichment of mitochondrial functions seen in transcriptome and proteomics data^71^
Eukaryotic translation elongation factor 1 gamma	EEF1G	Enrichment of gene transcription/translational processes, and ribosomal elements^72, 73^.
High mobility group box 2	HMGB2	Stress inducible protein and highly orthologous to known CD24-interacting protein HMGB1^46, 47, 74^.
Growth factor receptor bound protein 2	GRB2	Important downstream effector of B cell signaling^75^.

**Figure 4:**
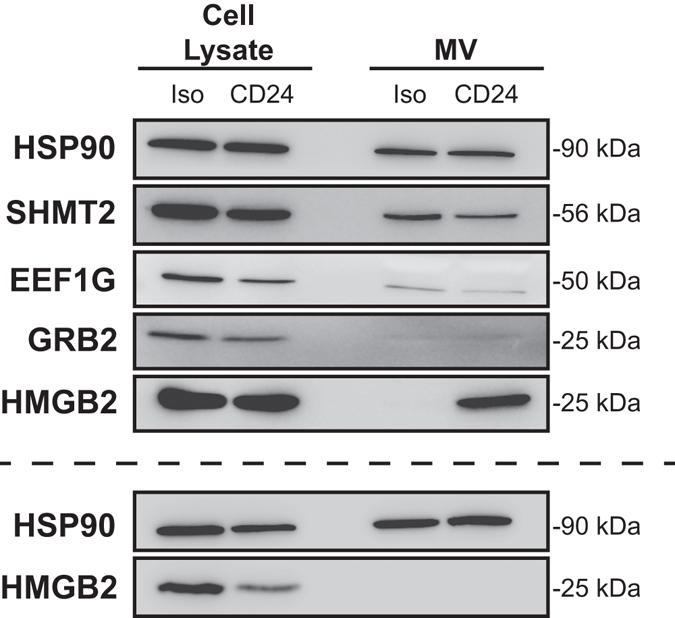
Proteins identified by proteomics analysis of B cell MVs are detectable in both cell lysates and MVs of isotype and anti-CD24 stimulated B cells by Western blot analysis. Cell lysates (5 µg; equivalent to approximately 2.3 × 10^5^ cells) and the corresponding protein from MVs (from 1.0 × 10^6^ cells) were analyzed for expression of SHMT2, EEF1G, GRB2 and HMGB2. HSP90 was used as a loading control after stimulation with isotype (Iso) or anti-CD24 (CD24). n = 5. Two different outcomes were observed for HMGB2, with MVs from CD24 stimulated cells containing high HMGB2 (Upper panels, n = 2) or HMGB2 being low/absent in all vesicles (lower panels n = 3).

By Western blot, we found that HSP90 is readily detectable and comparably expressed in all cell lysate and EV samples, and does not vary in response to anti-CD24 stimulation (Fig. 4). We easily detected SHMT2, EEF1G, HMGB2 and GRB2 in all cell lysates; however, GRB2 was detectable only at low level. The expression of these proteins in cell lysates was not affected by stimulation (Fig. 4). In contrast with our MS analysis, we observed SHMT2 and EEF1G in EVs from both isotype and anti-CD24 stimulated cells, with no difference in abundance in response to anti-CD24 stimulation. GRB2 was not detected in any EV sample, potentially due to its overall low abundance. Finally, there was considerable heterogeneity in HMGB2 in EVs, with two apparent phenotypes observed. In two replicates, HMGB2 was present in EVs from the anti-CD24 stimulated cells, but absent or minimally present in EVs from isotype stimulated cells, as predicted by MS. However, in three other replicates, HMGB2 was not detected in any EVs.

Overall, our proteomics analysis suggests that CD24 stimulation may enrich EVs with proteins involved with mitochondrial or metabolic functions. While MS analysis suggests specific protein enrichments following anti-CD24 stimulation, we were unable to confirm these enrichments via Western blot, likely due to differences in the sensitivity between the techniques, and intrinsic heterogeneity between EV samples.

### CD24 stimulation produces EVs with a distinct surface composition

We next examined surface protein expression on cells and EVs by FACS in response CD24 stimulation. Two receptors, Siglec-2 (CD22) and Siglec-G were selected based on their potential in acting as CD24 signaling partners^26–28^. CD63 was selected as a marker of EVs^29^. The B cell receptor (BCR), detected as IgM, is known to share downstream signalling pathways and synergize with CD24^14^. MHC-II was selected as a marker of B cell activation and previous associations with activated B cell exosomes^23^. Finally, Ter119 was selected as a putative negative control as its expression has been shown to be limited to erythroid-lineage cells^30^.

**Figure 5:**
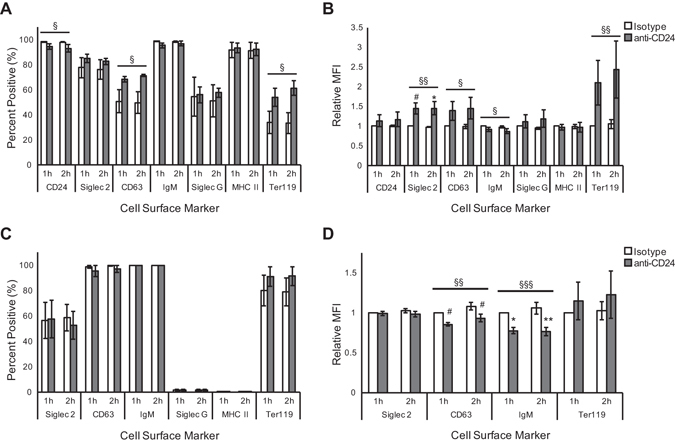
CD24 stimulation induces the formation of a unique B cell MV surface phenotype that does not reflect the cells from which they are released. Cells (**A**,**B**) and MVs (**C**,**D**) were analyzed for their expression of the indicated cell surface markers. Data are shown as mean ± SEM at 1 h or 2 h stimulation with either isotype or anti-CD24 Ab. n = 3–4. Cells or CD24-bearing MVs bound to beads were assessed for the percentage of (**A**) cells or (**C**) MVs positive for the individual markers, and (B/D) their relative mean fluorescent intensities (MFI). Significant differences were assessed by two-way ANOVA followed by Tukey post-hoc, if significant. Main effect of stimulation: ^§^P < 0.05, ^§§^P < 0.01. Significant changes compared to time-matched control: ^#^P < 0.1, *P < 0.05, **P < 0.01. Non-statistically significant changes have no indicators.

CD24 stimulation causes a statistically significant decrease in the number of CD24+ cells compared to isotype-treated cells after 1 h (Fig. 5A). No effect of stimulation time (1 h vs 2 h) was observed. The number of cells that expressed IgM, MHC-II, Siglec-2 and Siglec-G did not change in response to either Ab stimulation or time (Fig. 5A), while the number of cells expressing CD63 increased significantly in response to CD24 stimulation. We previously established that Ter119 is not expressed on isolated splenic B cells (data not shown) consistent with literature reports^30^, but unexpectedly, we found that it is expressed on WEHI-231 cells. Furthermore, significantly more cells express Ter119 in response to anti-CD24 stimulation.

There were also statistically significant changes in the relative abundance (measured by mean fluorescent intensity; MFI) of Siglec-2, CD63, Ter119 and IgM on the cell surface in response to anti-CD24 stimulation (Fig. 5B). The relative MFI of Siglec-2 and CD63 each increased by 1.4-fold at 1 h and 2 h post-stimulation. There was a 2.1-fold increase in Ter119 after 1 h, and a 2.4-fold increase after 2 h of CD24 stimulation. In contrast, there was a slight, but significant decrease in the relative MFI of IgM, as anti-CD24-stimulated cells had a relative MFI of 0.91 after 1 h and 0.87 after 2 h, compared with isotype-treated controls. Overall, the changes in MFI were due to the stimulation of CD24, but were independent of stimulation duration.

We have shown previously that EVs can transfer CD24 protein between cells^15^. Therefore, we analyzed the expression of the same proteins as above on the population of immunoaffinity-isolated, CD24+ EVs to determine if the surface composition differs in this population of EVs. This strategy also improves the detection of proteins on the overall EV population, as all EVs are measured even if they are too small to be analyzed by FACS.

We found that there were no statistically significant differences at 1 h or 2 h in the number of beads positive for IgM, Siglec-2, CD63 or Ter119 EVs from isotype or anti-CD24-stimulated cells (Fig. 5c). Essentially 100% of beads were positive for IgM (99.9 ± 0.1 %) and CD63 (97.7 ± 2.1%); however, only 56.3 ± 12.6% of the beads were positive for Siglec-2. While 79.4% of beads with EVs from isotype-stimulated cells and 91.2% of beads with EVs from anti-CD24-stimulated cells were positive for Ter119, this difference was not statistically significant. Surprisingly, we were unable to detect EVs containing Siglec-G or MHC-II from either condition, despite cells being positive for both.

While cells showed an increase in the MFI of Siglec-2 in response to anti-CD24 stimulation, no difference was observed in EVs (Fig. 5d), The MFI of CD63 on cells increased after anti-CD24 stimulation, which was opposite to the statistically significant decrease in CD63 MFI on the EVs at 1 and 2 h. Finally, we found that anti-CD24 stimulation induced significant decreases in the MFI of IgM on bead-captured EVs, which was similar in direction but not magnitude to the change seen with the cells.

Overall, we found that anti-CD24 stimulation can induce significant effects on the surface composition of cells and EVs. Furthermore, we found that select surface receptors are excluded from EVs, irrespective of cell stimulation, demonstrating that the membrane components of EVs do not necessarily reflect the bulk cell surface, but can be selected for via an unknown process.

## Discussion

Cells produce a variety of different extracellular vesicle subtypes, defined in part through their mechanism of biogenesis, and in part based on their morphology and composition^1^. As such, there is considerable overlap between population definitions, and no “gold-standard” exists to delineate vesicle sub-groups^31^. We have therefore followed recommendations to examine multiple EV components, including their RNA, representative membrane, cytosolic and intracellular proteins, as well as overall morphology to define the EV subtype we have isolated in this study^31^.

First, our analysis of the RNA and protein cargo carried by these B cell EVs is more consistent with reports on MVs rather than exosomes. While RNA profiling is relatively new for EVs, the RNA cargo carried by the EVs described here resembles that of MVs, but not exosomes or apoptotic bodies^18^. Specifically, the lack of 18S and 28S rRNA in the Bioanalyzer analysis, and confirmed by sequencing analyses, and the shorter length of the RNA transcripts is more consistent with MVs than with apoptotic bodies, or exosomes^18^. In the protein cargo, we identified the presence of several HSPs, including HSP90, and exploited the presence of membrane-integral HSPs to isolate EVs using the Vn96 peptide^17^. While HSPs are considered pan-EV markers in that they are present in most, if not all EVs, HSP90B1 has been reported to be under-represented in exosomes but present in other EV species^31^. Our proteomics analysis revealed that HSP90B1 is common to all EVs, regardless of condition. Our MS analysis also identified other proteins associated with intracellular compartments under-represented in exosomes, such as Calreticulin from the endoplasmic reticulum and Histone cluster 1h4a, providing further support that the EV population is not primarily composed of exosomes^31^.

Secondly, we detected the presence of several integral membrane cell surface receptors, including Siglec-2, IgM, and CD63 by FACS. While CD63 was previously considered an exosome marker, it is now appreciated that different EV species share compositional overlaps, and that this marker is not likely to be exclusive to exosomes^1, 32^. We also identified the presence of phosphatidylserine (PS) via Annexin V-staining, which is typically associated with MVs rather than exosomes^33^. We also found that these EVs lack MHC-II, known to be present on exosomes from activated B cells^23^.

Finally, through multiple techniques, including TEM, NTA and our previous FACS analysis, we have established that the EVs described here range in size from 70 nm to 170 nm. This size is on the larger end of sizes reported for exosomes, but in line with reports on MVs^2^. Our previous analysis suggested the EVs released from WEHI-231 cells in response to CD24 are plasma membrane derived (as are MVs) as there was no evidence of multivesicular body formation^15^. Taken together, these data strongly support that the majority of the EV population in this case is primarily comprised of MVs, and not exosomes or apoptotic bodies, and that CD24 stimulation promotes the genesis of additional phosphatidylserine-positive MVs.

**Figure 6:**
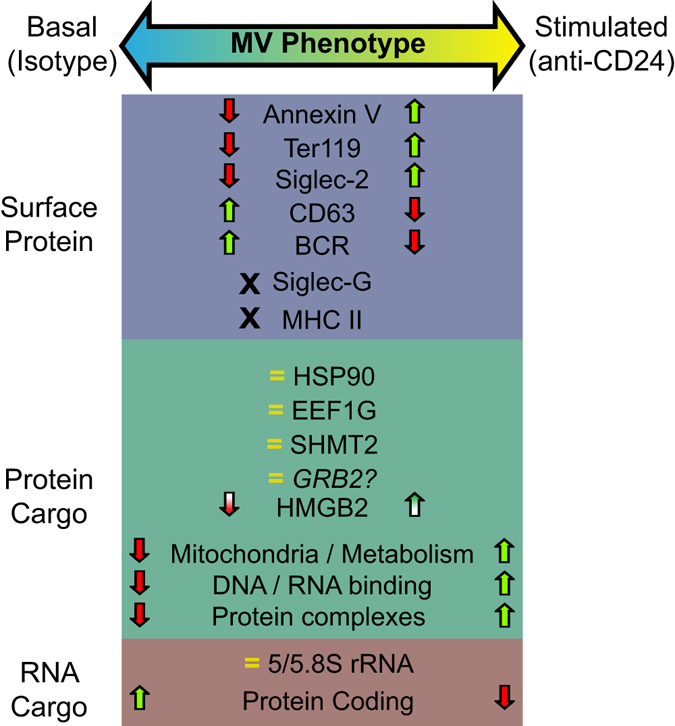
Summary diagram of the cargo and surface composition of MVs from isotype or CD24 stimulated cells. The differences in surface protein, luminal protein, and RNA transcriptome between basal and CD24 induced MVs are shown. No change indicated by=, absence indicated by X, a significant change indicated by arrows where arrows coloured with a gradient indicate a variable difference in abundance as detected by Western blot.

Within the cargo of these MVs, we observed considerable variability in MV RNA and protein composition. Our investigation suggests WEHI-231 B cells produce MVs with a spectrum of compositions following isotype and CD24 stimulation (Fig. 6). There are several potential reasons for this variability. First, vesicle packaging is limited by available lumen area. The interior space of exosomes (or similarly sized MVs such as described here) is calculated to be 20 nm to 90 nm^3^ ^34^. Thus, RNA and proteins compete for limited space. Our measurements of MV RNA, averaging under 5 ng of total RNA per isolation (data not shown), agree with other assessments and indicate that these MVs package very few transcripts per vesicle^35^. Thus, small differences in packaging, or skewing towards increased protein inclusion will lead to increased heterogeneity.

Secondly, cells can produce multiple vesicle populations simultaneously^32^, which may increase the variability of cargo observed when isolating total EV populations, as was performed here. This results in a heterogeneous release of differing cargo among EVs. For example, the colon cancer cell line LIM1863 simultaneously releases at least three distinct EV sub-populations that have a mosaic of common, and EV-subtype unique, miRNAs^36^. In addition, EV cargo sorting is non-binary and exists as a spectrum of efficiencies^37, 38^. Furthermore, EV subpopulations can be extensively variable in terms of their cargo (i.e. protease subunit inclusion), surface composition (such as phosphatidylserine inclusion), or the presence or absence of organelles^39^.

Studies using methods that select for a single EV sub-population (such as differential centrifugation, sucrose gradient floatation, or size-exclusion filtration) may exclude some EV populations and thus underestimate total EV cargo complexity. Additionally, the use of larger sample volumes^16, 40^ or highly enriched cell culture supernatants^16^ may also underestimate heterogeneity by reporting the dominant cargo species rather than capturing the population heterogeneity. In comparison, our analysis was on a small number of cells in a low volume of media with short duration of stimulation.

Finally, we detected the presence of HSPs, which can participate in protein degradation^41–43^, and multiple proteasome sub-units in all six of our MV samples. This suggests that these MVs may contain proteolytically active components, which could result in the degradation of packaged vesicle proteins. Moreover, this may explain our inability to detect some proteins by Western blot that were detected by MS, which relies on measurement of peptide fragments and not intact proteins.

The RNA cargo within the WEHI-231 B cell MVs was primarily composed of 5.8S and 5S rRNA. Since we did not detect the presence of 18S or 28S rRNA by Bioanalyzer or by sequencing, we believe that these MVs do not carry these components. This agrees with other MV analyses from human HMC-1 mast cells and mouse BV-2 microglia cells, which also lack 18S and 28S rRNA^18^. It is unknown what the functional consequence of this apparent differential selection of rRNA species may be.

These MVs also carry protein-coding transcripts from both nuclear and mitochondrial genes, with the majority encoding components of the electron transport chain; however, we did not identify any differentially included transcripts in response to CD24 stimulation. We did observe a reduction in the overall abundance of protein coding RNA and a trend towards increased miRNA incorporation following stimulation. Only two of the miRNAs were annotated: miR-6236 and miR-5099. There is little information on the function of these miRNAs; however, miR-5099 expression is associated with B cell development^44^ and binding to Argonaut, which is a key component in miRNA-induced RNA silencing in helper T cells^45^. Overall, the purpose of these transcripts and compositional changes in B cell MVs are unknown and may therefore be of interest in future studies on the biological function of these MVs.

Our proteomics analysis also suggested that CD24 may affect the protein cargo carried by WEHI-231 B cell MVs. We found there were 41 unique proteins, identified by MS, carried in all six MV samples. A substantial number of these were canonical HSPs, which is expected as the Vn96 peptide-mediated capture of EVs is known to depend at least partially on binding to HSPs, which are known to be enriched in EVs^1, 17^. As discussed above, a second major group of proteins identified in all six MV samples belong to the proteasome complex involved in protein degradation. While there were no unique proteins associated with MVs released following CD24 stimulation, there were 77 proteins identified as selectively enriched in at least 2 of 3 anti-CD24 stimulated MV samples.

Many proteins enriched in MVs from CD24 stimulated cells are involved in RNA shuttling, processing or stability. These include the KH-type splicing regulatory protein (Khsrp), Karyopherin (Importin) β-1 (Kpnb1), Ran binding protein 1 (Ranbp1), Heterogenous nuclear ribonucleoprotein A2/B1 (Hnrpa2b1), Poly(A) binding protein, cytoplasmic 1 (Pabpc1), and Poly(rC) binding protein 1 (PCBP1). These enriched proteins, along with the loss of protein coding mRNA and a trend towards increasing miRNA incorporation into MVs from CD24-stimulated cells, suggest a mechanism by which select miRNA transcripts could be enriched in MVs and influence recipient cell behaviour through their MV-mediated delivery and subsequent effect on the gene expression profile of the recipient cells. Future studies are planned to examine how the RNA and protein cargoes of these MVs could influence recipient cell behaviour.

When we attempted to validate the CD24-mediated changes in protein cargo using four different proteins, representing different biological functions, we found that while all the proteins were present in the cells, only EEF1G, SHMT2, and HMGB2, but not GRB2, were detectable in the EVs; however, the abundance of EEF1G and SHMT2 is not altered with CD24 stimulation. Interestingly, we found that HMGB2 had variable inclusion into MVs from CD24-stimulated cells, with it being highly enriched in MVs in response to CD24-stimulation in two biological replicates, but undetectable in MVs in additional replicates, regardless of the stimulation. We do not believe that this is a technical error, as other MV proteins (such as HSP90) were easily detected in the same replicate and there were very high levels of HMGB2 when it was detected. HMGB2 is closely related to HMGB1 and HMGB3, which are danger-associated molecular patterns that act in response to cellular stress^46, 47^. Thus, we believe that there may be an additional stress event, which we have not yet identified, that allow HMGB2 to be released in response to CD24 stimulation. Studies are ongoing to identify additional signals that regulate HMGB2 release.

Overall, regardless of stimulation, a significant contribution to the MV RNA and protein cargo was related to mitochondrial components and functions. This included mitochondrial transcripts and proteins involved in metabolite generation and electron transport activity. We therefore propose these MVs, regardless of CD24 stimulation, are associated with proper mitochondrial maintenance. As with other cancers, B cell leukemia or lymphoma cells (such as WEHI-231 cells used here) show increased markers of oxidative stress and reactive oxygen species (ROS) formation^48^. Increased ROS production can negatively affect cell viability, leading to increased caspase activation, and ultimately cell death^49^. As CD24 stimulation in B cells also results in caspase activation leading to apoptosis^13^, the additional stress from CD24 stimulation may compound the challenge of dealing with ROS in these cells. Thus, the release of mitochondria components in WEHI-231 B cell MVs, and the enrichment of these components following CD24 stimulation, may act as a mechanism to regulate mitochondrial health. This is consistent with a recently-described process termed mitoptosis, which involves the selective sequestering, destruction and disposal of dysfunctional mitochondria to mitigate ROS stress and preserve cell viability^50^. During mitoptosis, these dysfunctional mitochondria may be selectively discarded through plasma membrane blebbing (such as occurs in MV formation) in vesicles 50 nm to 200 nm in size^50^. Therefore, the association we report here between CD24 stimulation and an increase in the release of MVs enriched in mitochondrial components and cell stress markers (like HMGB2) may be related to mitoptosis, or a similar process, to regulate cell health and viability. Future studies on the association between CD24-stimulated B cell EV release and B cell health will be necessary to examine this possibility. As B cell development and activation is delineated into many distinct sub-types, with differing levels of CD24 expression, it will be important to determine how different B cell developmental stages, or even disease states, respond to CD24 stimulation with respect to EV generation.

Despite the variability in RNA and protein cargo, we observed clear phenotypic differences with respect to the cell surface receptor composition of cells and EVs, and that CD24 can induce specific changes to this composition. Approximately 50% of cells were positive for Siglec-G, regardless of stimulation, and all were positive for MHC-II; however, MVs carried neither protein. As both these proteins are integral membrane proteins, they must be excluded from the membrane domain from which MVs are released. In addition, following CD24 stimulation, cells increased their relative expression of Siglec-2 (CD22) but this was not reflected in the MVs. Similarly, CD24-stimulation caused an increase in the percentage of cells expressing CD63 from approximately 50% to 68.3% at 1 h and to 71.5% at 2 h, as well as increasing its relative abundance. In contrast, nearly 100% of the MV populations from both conditions expressed CD63, and CD24 stimulation caused a *decrease* in the relative abundance of CD63 carried by MVs. These observations clearly demonstrate that MVs are not merely representative of the cells from which they originate, and that aspects of their surface composition must be regulated during formation.

The release of exosomes by B cells is associated with their activation during immune signalling^51, 52^. Our analysis suggests the MVs isolated here are not related to WEHI-231 B cell activation. First, B cell activation requires BCR stimulation, which was not performed here. Also, Siglec-2 is antagonistic to BCR signalling^53^, thus the observed increase in Siglec-2 expression would be expected to inhibit BCR signalling. Additionally, B cell exosomes carry antigen-presenting MHC-II^23^ whereas we found no MHC-II expression on the MVs captured. Therefore, the distinct surface composition, as well as the abundance of mitochondrial contents, argues these MVs are formed and released to perform a distinct function compared with exosomes released during B cell activation. The functional consequences of these compositional differences are unknown, but will likely be an important consideration in understanding the function of exosomes compared to MVs.

While it is now clear that CD24 can induce changes in WEHI-231 B cell MV composition, a functional role of CD24 itself has yet to be identified. Several studies propose that CD24 may be useful as a marker of EVs^16, 17^; however, its function has not been elucidated. One potential role may be to mediate cell-MV recognition to promote uptake by recipient cells, since CD24 can also mediate cell adhesion events through cell-specific ligands, such as selectins^54–56^. It was recently shown that Siglec-1 (CD169) is required for exosome capture by macrophages and apoptotic vesicle recognition by immune cells through binding to proteins modified with α2,3 sialic acid on the surface of EVs^57, 58^. CD24 is also modified by the addition of α2,3- and α2,6 sialic acids, thus it is possible that the α2,3 and/or α2,6 sialic acids on CD24 promote interaction of EVs with Siglec-expressing cells. Siglec-G, which is structurally similar to Siglec-1^27, 59^, was retained on the B cell surface rather than incorporated into their MVs, suggesting that B cells may be selectively retaining receptors capable of interacting with these CD24-bearing MVs.

The ability of CD24 to promote MV cargo composition changes likely affects the function of these MVs, and the specific changes in surface receptors on the MVs may influence their intended extracellular targets. Compositionally, the mitochondrial cargo carried by these MVs, and potentially enriched by CD24, suggest they may have a role in regulating mitochondrial health or stress. This study represents the first comprehensive analysis of EVs released following CD24 stimulation. Future studies will be needed to determine if EV heterogeneity and CD24-mediated regulation of EV surface protein composition is generalizable to other B cell lymphomas, healthy B cells, or other CD24-expressing cells.

## Materials and Methods

### Cell Culture

Cell culture materials were obtained from Thermo Fisher Scientific (San Jose, CA) unless otherwise indicated. The WEHI-231 pre-B cell lymphoma cell line (ATCC; Manassas, VA) was maintained in RPMI 1640 media supplemented with 10% heat-inactivated fetal bovine serum (FBS), 1% penicillin and streptomycin, 1 % sodium pyruvate and 0.1% β-mercaptoethanol (RPMI complete) at 37 °C and 5% CO_2_.

### EV Production

#### Vesicle-free media

Two aliquots of RPMI complete media were prepared as described^60^, with the following changes: 20% heat-inactivated FBS (RPMI-20%) was centrifuged at 100,000 × g for 18 h at 4 °C in an SW-28 rotor (Beckman Coulter, Brea, CA) to deplete endogenous vesicles from the FBS, and filtered through a 0.22 µm filter and stored at 4 °C. Vesicle-free RPMI for culturing was prepared by mixing equal volumes of vesicle-free RPMI-20% and FBS-free RPMI-complete media.

#### Stimulation of EV production

For stimulation and vesicle collection, 5.0 × 10^5^ WEHI-231 cells were removed from RPMI-complete, washed 1X in vesicle-free RPMI, re-plated in 1 mL vesicle-free media and rested for 5 min at 37 °C. Cells were then stimulated at 37 °C with either 10 µg/mL functional grade primary (1°) isotype control Ab (cat no. 16-4031-85) or M1/69 rat anti-mouse CD24 Ab (cat no. 16-0242-85) from eBioscience (San Diego, CA), which had been pre-incubated with 5 µg/ml goat anti-rat secondary (2°) Ab (Jackson ImmunoResearch; West Grove, PA) for 10 min at room temperature. Either unconjugated (cat no. 112-005-003) or biotinylated (112-065-003) 2° Ab was used depending on the subsequent analysis. These stimulations are referred to as isotype and anti-CD24, respectively. Isotype antibody has previously been demonstrated to not bind to WEHI-231 cells^15^ and is used in place of an unstimulated control.

### Nanoparticle Tracking Analysis

WEHI-231 cells were stimulated as described using biotinylated 2° Ab to remain consistent with previous Ab stimulations. Following stimulation, the cells were centrifuged at 500 × g for 5 min to pellet cells and then centrifuged at 2000 × g for 5 min to pellet cell debris and larger vesicles. Conditioned media from isotype-treated or anti-CD24-treated WEHI-231 cells were diluted 1:25 in 0.1-μm-filtered PBS and immediately analyzed on an LM10 Nanosight system with software version 3.2 (Malvern; UK). Five videos of 30 sec each were acquired using camera level 15 for all samples as well as a background media control. The quintuplicate videos for each sample were batch analyzed using a detection threshold of 10. Mean number of particles/ml for each batch was used to estimate the original concentration.

### Isolation of Extracellular Vesicles (EVs)

#### Immunoaffinity isolation

WEHI-231 cells were stimulated as described using biotinylated 2° Ab. Following stimulation, the cells were centrifuged at 500 × g for 5 min to pellet cells. Cells were stained for FACS as described below as needed. Supernatant containing vesicles was then centrifuged at 2000 × g for 5 min to pellet cell debris and larger vesicles. Protease and phosphatase inhibitors (1 mM phenylmethylsulfonyl fluoride (PMSF; Sigma-Aldrich, St. Louis MO), 1 mM sodium orthovanadate (Sigma-Aldrich) and 1 µM aprotinin (Sigma-Aldrich)) were added to the supernatant. Anti-CD24 M1/69 (10 µg/mL) and biotinylated 2° Ab (5 µg/mL) were added to supernatant from istotype-treated cells. Supernatant (1 ml) was then incubated with 2.2 × 10^6^ streptavidin-coated magnetic beads (average diameter 4.0 µm; Spherotech; Chicago IL) pre-blocked in 5% bovine serum albumin (BSA) in phosphate-buffered saline (PBS) with rotation, overnight at 4 °C. Beads and the bound EVs were then isolated using an EasySep magnetic separation system (StemCell; Vancouver, Canada) followed by FACS analysis (see below).

#### Vn96 peptide-based isolation

WEHI-231 cells were stimulated as described using unconjugated 2° Ab. Supernatant (1 ml) and cells were collected, centrifuged and treated with protease inhibitors as above. Vn96 peptide (22.5 µg) was suspended in 9 µL ME-buffer (New England Peptide; Gardner MA) and added to approximately 750 µL of cleared supernatant. Vn96 was incubated with supernatant with rotation, overnight at room temperature. Vn96 with bound EVs was pelleted by centrifugation at 17,000 × g for 15 min at room temperature, producing a translucent pellet, following the manufacturer’s instructions. No pellet was produced from the supernatant in the absence of Vn96. The pellet was further enriched for EVs by adding a second aliquot (750 µL) of vesicle-containing RPMI-media, followed by disrupting the pellet using a 1000-µL pipette and incubating with rotation for 1 h at room temperature. Vn96-EV were isolated again by centrifugation at 17,000 × g for 15 min. Pellets were washed once in 0.1 µm-filtered PBS and centrifuged at 16,000 × g for 10 min. Vn96-EV pellets were resuspended in buffers appropriate for the subsequent analysis as described below.

### Transmission Electron Microscopy

EVs were isolated using Vn96 from supernatant from isotype and anti-CD24 treated cells as described above. Two 750-µL aliquots of vesicle-containing media were pooled for each Vn96 pull-down. Pellets were resuspended in 20 µL of PBS and MV were dispersed by digestion overnight with 25 µg proteinase K enzyme^17^ at 37 °C. The digested samples were centrifuged at 17,000 × g for 15 min to remove undigested Vn96-EV material. Dispersed EV (10 μl) were placed on formvar-carbon electron microscope grids (Canemco; Montreal, Canada) and allowed to dry for 30 min. Grids were floated sample-side down pyrogen-free water. Grids were then fixed with 3.7% paraformaldehyde for 15 min, followed by two washes with water by flotation. Grids were contrasted with 2% uranyl acetate (w/v), followed by one additional water wash. All solutions were filtered using 0.1-µm syringe filters (4611; Pall Corp; Port Washington, NY). Dried grids were then viewed using a Tecnai Biotwin Transmission Electron Microscope (TEM) (FEI; Hillsboro OR). Images were captured using an XR-41 camera with an AMT capture engine V602 (Advanced Microscopy Techniques; Woburn, MA).

### Transcriptome Analysis

#### RNA sequencing

All sequencing materials and equipment are from Thermo Fisher Scientific unless otherwise noted. Vn96-EV pellets were resuspended in 1 mL of TRIzol reagent. Following RNA extraction, RNA quantity was measured by Nanodrop spectrophotometer (Thermo Fisher Scientific). RNA samples were then assessed using the Agilent Bioanalyzer with the RNA 6000 Pico kit (Agilent; Santa Clara CA). Using RNA concentration values from the Bioanalyzer, samples were prepared for sequencing on the IonTorrent PGM per the manufacturer’s protocol. In brief, all recovered MV RNA was concentrated to 3 µL by vacuum centrifugation. Library preparation was performed, without RNA fragmentation, following the guidelines for the IonTotal RNA-Seq Kit v2. Barcode adapters were used in the cDNA amplification process to differentiate isotype (barcode 1) from anti-CD24 (barcode 2) stimulated cells. Amplified cDNA was enriched using the Ion PGM Template OT2 200 Kit and the Ion OneTouch 2 System. Library density on the IonSphere particles was assessed via Qubit (Thermo Fisher Scientific) fluorometric quantitation. Libraries were then loaded onto an IonTorrent 316 v2 Chip and sequenced for 550 reads.

#### Bioinformatics analysis

IonTorrent transcript read counts were pre-processed as per Anders *et al*. (2013). For quality control checks, we used the FastQC software (http://www.bioinformatics.babraham.ac.uk/projects/fastqc/). Read trimming was performed with the FastQ quality trimmer as part of the FASTX toolkit (http://hannonlab.cshl.edu/fastx_toolkit/) using the parameters –Q 33, -t 22, -I 28. Read mapping was performed by tmap with parameters –B 18, -a 2, -v stage 1, map1, map2, map3. For feature counting, we used the ht-seq-count script available in the HTSeq framework (version 0.5.4p5) with parameters–mode = union–stranded = yes–type = exon–idattr = gene_id -a 0 and the genome annotation downloaded from Ensembl (dataset mmusculus_gene_ensembl, release GRCm38.p5)^61^. Analysis of sequencing data was performed using R 3.3.1^62^ accessed by RStudio 0.99.902^63^. RNA annotation was performed using BioMart^19^. Differential gene expression analysis was performed using edgeR following the protocol describe in Anders, *et al*.^61, 64^ with Counts Per Million filters of 1.0 in 3 or more replicates.

### Proteomics

#### Protein separation and in-gel tryptic digests

The EV-Vn96 complexes (pellets) were re-suspended in 25 μL of PBS followed by addition of 2X Laemmli buffer containing β-mercaptoethanol (Biorad; Hercules CA), heated for 5 min at 95 °C and then stored at −20 °C. Protein mixtures (45 μL) were separated on a 10% SDS-PAGE gel and visualized with Coomassie EZBlue stain (Sigma-Aldrich). Each gel lane was excised into 12 bands that were distributed into individual micro-centrifuge tubes for tryptic digestion. Each of the bands was sequentially treated with 10 mM dithiothreitol (Sigma-Aldrich) and 25 mM iodoacetic acid (Sigma-Aldrich) to reduce internal disulfide bonds and alkylate free cysteine residues, respectively. Fifty μL of a 10 ng/μL solution of modified trypsin (Promega; Madison, WI) in 100 mM ammonium bicarbonate (Sigma-Aldrich) was added to each tube for overnight enzymatic digestion. The extraction of peptides was achieved using 50% acetonitrile (VWR; Mississauga, ON) containing 5% acetic acid (Sigma-Aldrich,). The total volume of each sample was reduced by vacuum centrifugation to approximately 45 μL, adjusted to 1% acetic acid and stored at −80 °C.

#### Offline C-18 solid-phase extraction

Peptide extracts were prepared for offline C-18 clean-up by adding 2% formic acid (Sigma-Aldrich)/20% ACN to each sample at a ratio of 1:3. C-18 mini spin-filter cartridges (Canadian Life Science; Peterborough, ON) were initially activated with 50% ACN and then equilibrated with a 0.5% formic acid/5% ACN solution. Extracted protein digests were bound to the C-18 resin, washed with 0.5% formic acid/5% ACN, and eluted from each filter with a 70% ACN solution. Sample volumes were then reduced by vacuum centrifugation to 45 μL and adjusted to 0.1% aqueous formic acid.

#### Mass spectrometry analysis

Mass spectrometric protein identification data were analyzed using Proteome Discoverer version 1.4 (Thermo-Fisher Scientific) employing the SEQUEST scoring algorithm^65^. FASTA databases were obtained from Uniprot^66^ for *Mus musculus* (44,435 kb) and from the Global Proteome Machine for contaminants using the contaminant repository for affinity purification (cRAP) entries (41 kb)^67^.

Peptide identifications were accepted if they could be established at greater than 95.0% probability by the Scaffold Local FDR algorithm^68^. Protein identifications were accepted if they could be established at greater than 99.0% probability and contained at least 2 unique identified peptides. Protein probabilities were assigned by the Protein Prophet algorithm^68^. Proteins that contained identical peptides were grouped to satisfy the principles of parsimony.

### Ontology Enrichment Bioinformatic Analysis

Transcriptomics and proteomics data were assessed to identify enriched gene ontologies (GO) using the ToppFun program in the ToppGene suit^69^ using official gene symbols. P-values were obtained using the probability density function. Bonferroni multiple testing correction (q-value) was performed and results were reported with q < 0.05. Association analysis was performed using default settings. The proteomics GO terms for common and CD24-enriched MV proteins were compared using the Venn diagram tool from the Bioinformatics Institute Ghent (http://bioinformatics.psb.ugent.be/webtools/Venn/). GO terms were grouped into common categories using the online Revigo tool^70^ using the associated Bonferroni FDR values from Toppfun. Ontology semantic similarity was measured using the SimRel function (Revigo default).

### Western Blotting

#### Cells

Cells were stimulated with either isotype or anti-CD24 Ab for 1 h. Cells were pelleted and resuspended in 100 µL of Tris-based RIPA lysis buffer (50 mM Tris-HCl; pH 7.6, 0.02% sodium azide, 0.5% sodium deoxycholate, 0.1% SDS, 150 mM NaCl) supplemented with 1 mM PMSF (Sigma-Aldrich), 1X HALT protease inhibitor cocktail (Thermo Fisher Scientific) and 1 µM aprotinin (Sigma-Aldrich). Cells were lysed for 10 min on ice, then centrifuged at 17,000 × g for 10 min to pellet cell debris. Protein was quantified with the Bicinchoninic Acid Protein Assay (Thermo Fisher Scientific) per the manufacturer’s protocol. Cell lysates were then prepared in SDS sample buffer (62.5 mM Tris base, 2% glycerol, 2.3% SDS, 100 mM dithiothreitol, 0.02% bromophenol blue, pH 6.8) and boiled for 5 min. Protein (5 μg) was loaded onto a 12% SDS-PAGE gel followed by transfer to nitrocellulose membrane. Blocking was performed using 5% (w/v) skim milk in TBST. Anti-mouse Ab were diluted in tris-buffered saline with 0.05% Tween-20 (TBST) + 5% BSA as follows: 1:1000 HSP90α/β (SC-13119; Santa Cruz; Santa Cruz CA), 1:750 SHMT2 (12762 S; Cell Signaling Technology; Danvers, MA), 1:1500 EEF1G (ab72368; Abcam; San Francisco CA), 1:1000 GRB2 (3972S; Cell Signaling Technology) and 1:1500 HMGB2 (14163S; Cell Signaling Technology). HSP90 was detected using goat-anti-mouse IgG (SC-2005; Santa Cruz) and all others were detected using goat-anti-rabbit IgG (SC-2004; Santa Cruz) Secondary antibodies were diluted 1:2000 in TBST + 5% BSA. Immobilon Western chemiluminescent horse radish peroxidase substrate (Millipore; Billerica MA) was used for detection. Images were acquired using the AlphaImager Gel documentation system with FluorChem HD2, v3.4.0 (Protein Simple; San Jose CA). Image manipulation was limited to adjusting brightness and contrast to the entire image.

#### Extracellular vesicles

EVs were isolated using Vn96 from isotype and anti-CD24 Ab treated cells as described above. Two 1-mL aliquots of vesicle-containing media were pooled for each Vn96 pull-down representing the total 1 h vesicle production from 1.0 × 10^6^ cells. Vn96-EV pellets were dissolved in SDS loading buffer and boiled for 5 min. Half of each Vn96-EV sample was loaded onto each 12% SDS-PAGE gel. Proteins were transferred, probed and detected as described for cells.

### Flow Cytometry

A FACSAria II SORP cell sorter was used to collect flow cytometry (FACS) data using FACSDiva v8.0 software (BD Biosciences; San Jose CA), at the Cold-Ocean Deep-Sea Research Facility (Memorial University of Newfoundland). Data analysis was performed using FlowJo v10.0.5 (Tree Star; Ashland, OR.). All reagents were from eBioscience (San Diego, CA) and are rat anti-mouse antibodies, unless otherwise stated. Colour compensation was performed using single-stained OneComp beads (01–1111) with all fluorophores. Cells were stimulated to produce EVs as described above using 1° Ab pre-incubated with biotinylated 2° Ab. Isotype or anti-CD24-treated cells were stained with 0.5 µg M1/69 CD24-FITC (11–0242) or Streptavidin-FITC (11–4317), respectively. All cells were stained with 1.25 µg Siglec-2-PE (126111; Biolegend; San Diego CA), 0.625 µg CD63 PerCP-eFluor710 (46–0631), 1.25 µg IgM PE-Cy7 (25–5790), 0.625 µg Siglec-G APC (17–5833), MHC-II (I-A/I-E) Alexa Fluor 700 (56–5321), and 0.625 µg Ter119 APC-eFluor780 (47–5921). Matching isotype Ab controls were used to confirm the absence of non-specific Ab binding and to set thresholds. Captured MVs were stained with the same fluorophores except with 5 µL Annexin V Alexa488 (Thermo Fisher Scientific) instead of anti-CD24-FITC or Streptavidin-FITC.

#### Cells

Cells were suspended in phosphate buffered saline (PBS; 1.86 mM NaH_2_PO_4_·H_2_O, 8.41 mM Na_2_HPO_4_, 150 mM NaCl) that contained 1% heat-inactivated fetal bovine serum (FACS buffer) unless stated otherwise. Pelleted cells were washed at 4 °C in 500 µL FACS buffer and resuspended in 100 µL of FACS buffer containing FITC-conjugated streptavidin, and the directly conjugated Siglec-2 (CD22), CD63, IgM, Siglec-G, MHC-II and Ter119 Ab diluted as above. Cells were stained for 30 min at 4 °C, followed by the addition of 500 µL of FACS buffer. Cells were again washed in 500 µL FACS buffer prior to fixation in 100 µL of 4% paraformaldehyde for 20 min at room temperature in the dark. FACS buffer (400 μl) was added to cells, which were then assessed on the FACSAria II. For analysis, single cells were gated using the FSC/SSC parameters.

#### Extracellular vesicles

EVs were isolated by immunoaffinity isolation. Following magnetic isolation, bead- bound MVs were washed in 1X Annexin V binding buffer (10 mM HEPES buffer, 140 mM NaCl, 2.5 mM CaCl_2_; pH 7.4). Bead-MV complexes were resuspended in 100 µL of Annexin V binding buffer containing the directly conjugated Siglec-2, CD63, IgM, Siglec-G, MHC-II and Ter119 Ab diluted as above and incubated for 20 min at room temperature. Samples were diluted with 400 µL of 1X Annexin V binding buffer and placed at 4 °C until analyzed the same day on the FACSAria II. Analysis was performed on singlet beads, gated using FSC/SSC parameters. Non-specific antibody binding to beads was established with pre-blocked beads, which were used to set the negative fluorescence threshold for all MV fluorescence parameters.

### Statistical analysis

All statistics were performed using R 3.3.1^62^ accessed by RStudio 0.99.902^63^. Two-way comparisons were assessed by two-tailed Students’ paired t-test with significance assessed as a p-value < 0.05. Multi-parameter comparisons (i.e. flow cytometry of surface characteristics) was assessed by two-way analysis of variance (ANOVA) followed by Tukey post-hoc test, if significant. Significance was defined as a Tukey-corrected p-value of 0.05 or lower. In all cases, results with p-values approaching significance, defined as p < 0.1, but not meeting the significance threshold of 0.05 were noted with an alternate symbol. Relevant tests are indicated within figure legends, and/or in the appropriate results sections, as noted.

### Data Availability

All RNA-Seq gene expression data have been deposited in the Gene Expression Omnibus (GEO) repository under accession number GSE94778. The mass spectrometry proteomics data have been deposited to the ProteomeXchange Consortium via the PRIDE partner repository with the dataset identifier PXD005919.

## Electronic supplementary material

Supplementary Information

Supplemental File 1

Supplemental File 2
