# Supplementary material for: CD24 induces changes to the surface receptors of B cell microvesicles with variable effects on their RNA and protein cargo
Source: Sci Rep. 2017 Aug 17;7:8642. doi: 10.1038/s41598-017-08094-8 (PMC5561059; doi:10.1038/s41598-017-08094-8)
Supplement: Supplementary file 1 — Supplementary Information [file 41598_2017_8094_MOESM1_ESM.pdf]

CD24 induces changes to the surface receptors of B cell microvesicles with variable effects on their RNA and protein cargo

D. Craig Ayre[1], Ian C. Chute[2], Andrew P. Joy[2], David A. Barnett[2], Andrew M. Hogan[1], Marc P. Gruell[3], Lourdes Peña-Castillo[3,4], Andrew S. Lang[3], Stephen M. Lewis[2,5,6,7] and Sherri L. Christian[1*]

[1]Department of Biochemistry, Memorial University of Newfoundland, St. John's, Newfoundland and Labrador, Canada

[2]Atlantic Cancer Research Institute, Moncton, New Brunswick, Canada

[3]Departments of Biology, and [4]Computer Science, Memorial University of Newfoundland, St. John's, Newfoundland and Labrador, Canada

[5]Department of Microbiology & Immunology, Dalhousie University, Halifax, Nova Scotia, Canada

[6]Department of Biology, University of New Brunswick, Saint John, New Brunswick, Canada

[7]Department of Chemistry & Biochemistry, Université de Moncton, Moncton, New Brunswick, Canada

Supplemental data

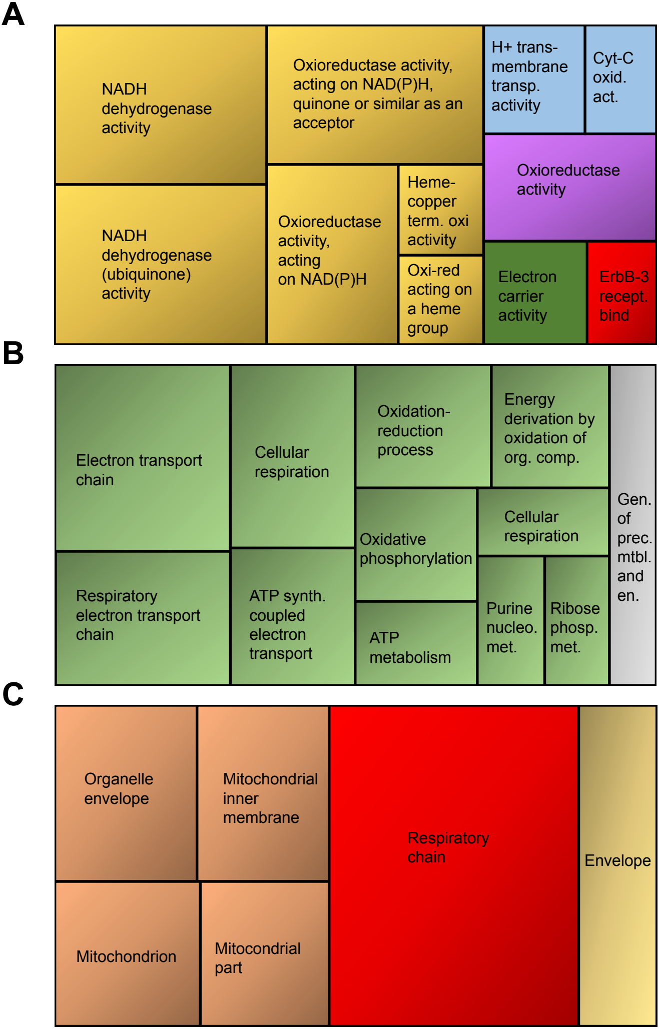
A
NADH dehydrogenase activity
Oxioreductase activity, acting on NAD(P)H, quinone or similar as an acceptor
H+ trans-membrane transp. activity
Cyt-C oxid. act.
Oxioreductase activity
NADH dehydrogenase (ubiquinone) activity
Oxioreductase activity, acting on NAD(P)H
Heme-copper term. oxi activity
Oxi-red acting on a heme group
Electron carrier activity
ErbB-3 recept. bind
B
Electron transport chain
Cellular respiration
Oxidation-reduction process
Energy derivation by oxidation of org. comp.
Gen. of prec. mtbl. and en.
Cellular respiration
Oxidative phosphorylation
Respiratory electron transport chain
ATP synth. coupled electron transport
ATP metabolism
Purine nucleo. met.
Ribose phosp. met.
C
Organelle envelope
Mitochondrial inner membrane
Respiratory chain
Envelope
Mitochondrion
Mitocondrial part

Supplemental Fig. 1: Revigo analysis of Gene Ontologies of RNA Cargo

The 50 most abundant protein coding RNA transcripts from isotype or anti-CD24 Ab stimulated cells are predominantly associated with mitochondrial function GO terms. No differentially expressed transcripts were identified in response to the different Ab treatments. (A) Biological processes (BP): NADH dehydrogenase (ubiquinone) activity (yellow), hydrogen ion transmembrane transporter activity (blue). (B) Molecular functions (MF): Electron transport chain (green). (C) Cellular components: Mitochondrial inner membrane (pink). Act. = activity, comp. = compounds, Cyt-c = cytochrome C, gen. = generation, en. = energy, mtbl. = metabolites, nucelo. = nucleotide, org. = organic, oxi = oxidase, oxi-red = oxioreductase, phosp. = phosphate, prec. = precursor, recept. = receptor, Transp. = transport, term. = terminal

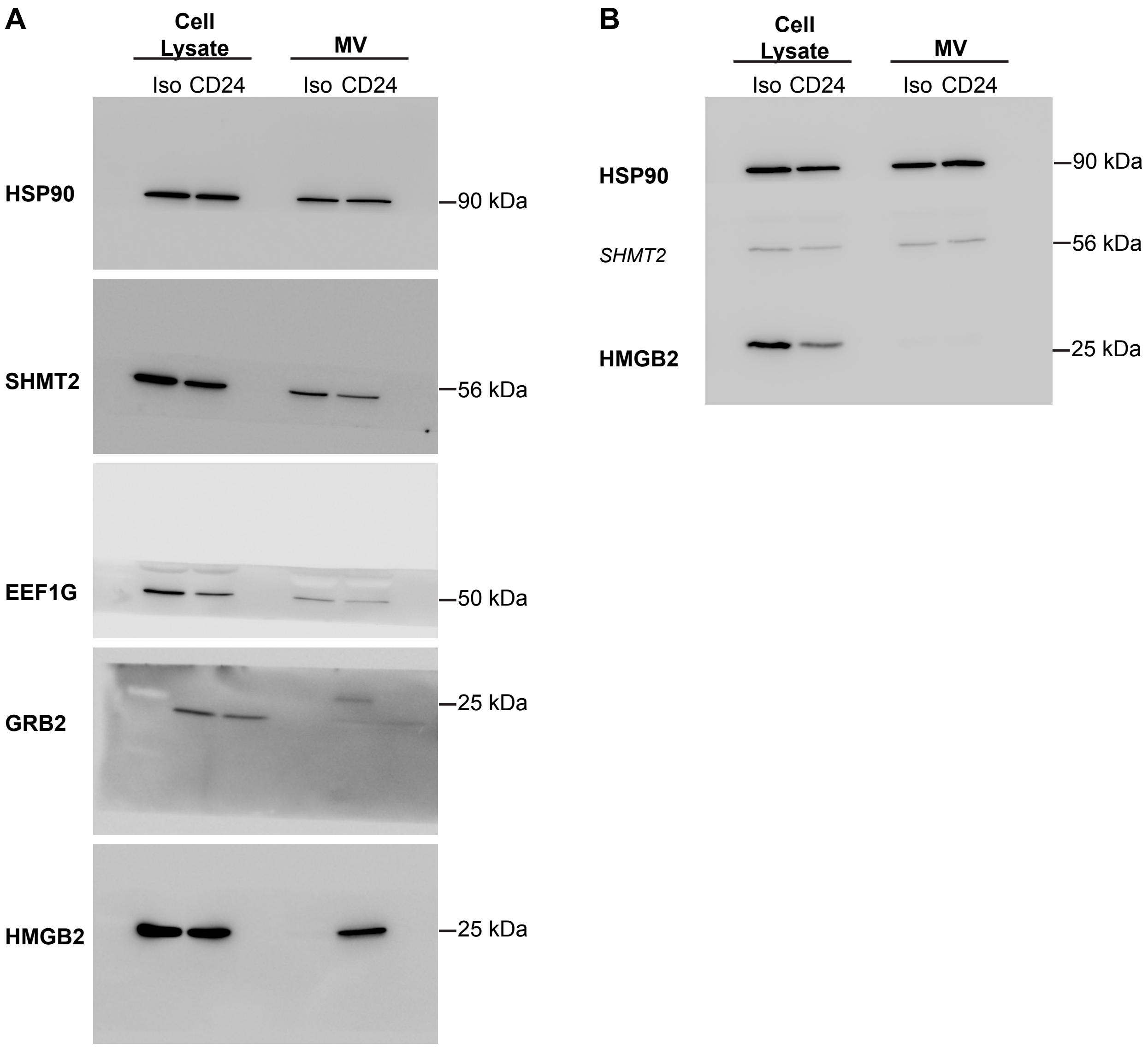
A
Cell Lysate
MV
Iso CD24
Iso CD24
HSP90
—90 kDa
SHMT2
—56 kDa
EEF1G
—50 kDa
GRB2
—25 kDa
HMGB2
—25 kDa
B
Cell Lysate
MV
Iso CD24
Iso CD24
HSP90
—90 kDa
SHMT2
—56 kDa
HMGB2
—25 kDa

Supplemental data: Western blot gels

Uncropped images of western blots presented in the main manuscript as figure 4. Irrelevant lanes used for another experiment that were prepared and run on these gels, but not part of this study, have been digitally removed. Membranes were cut immediately after transfer at the 70 kDa and 35 kDa markers to produce 3 membrane sections, which were probed individually for the proteins of interest, as appropriate for their molecular weights. A) Gel images for the upper 5 bands (HMGB2 present) in figure 4. B) Gel image for lower 2 bands (HSP90 and HMGB2) in figure 4. Also included in this gel image was SHMT2, which was not presented as part of the final figure. Molecular weight marker positions are shown to the right of each image.

Supplemental Excel File 1: Unique transcripts and proteins identified by RNA sequencing or MS/MS analysis of vesicles, respectively, from either isotype or anti-CD24 stimulated cells.

Supplemental Excel File 2: Ontological enrichment analysis of the top 50 protein coding transcripts identified by RNA sequencing of MVs from either isotype or anti-CD24 stimulated cells, and from proteins enriched in MVs from anti-CD24 stimulated cells.
